# An Overview of LEDs’ Effects on the Production of Bioactive Compounds and Crop Quality

**DOI:** 10.3390/molecules22091420

**Published:** 2017-08-27

**Authors:** Md. Mohidul Hasan, Tufail Bashir, Ritesh Ghosh, Sun Keun Lee, Hanhong Bae

**Affiliations:** 1 Department of Biotechnology, Yeungnam University, Gyeongsan, Gyeongbuk 38541, Korea; mhasan@hstu.ac.bd (M.M.H.); tufail.bashir1@gmail.com (T.B.); riteshghosh08@gmail.com (R.G.); 2 Division of Forest Insect Pest and Diseases, Korea Forest Research Institute, Seoul 02455, Korea; lskyou0425@gmail.com

**Keywords:** light-emitting diode, bioactive compounds, nutrition, antioxidant, fruit decay, disease resistance

## Abstract

Light-emitting diodes (LEDs) are characterized by their narrow-spectrum, non-thermal photon emission, greater longevity, and energy-saving characteristics, which are better than traditional light sources. LEDs thus hold the potential to revolutionize horticulture lighting technology for crop production, protection, and preservation. Exposure to different LED wavelengths can induce the synthesis of bioactive compounds and antioxidants, which in turn can improve the nutritional quality of horticultural crops. Similarly, LEDs increase the nutrient contents, reduce microbial contamination, and alter the ripening of postharvest fruits and vegetables. LED-treated agronomic products can be beneficial for human health due to their good nutrient value and high antioxidant properties. Besides that, the non-thermal properties of LEDs make them easy to use in closed-canopy or within-canopy lighting systems. Such configurations minimize electricity consumption by maintaining optimal incident photon fluxes. Interestingly, red, blue, and green LEDs can induce systemic acquired resistance in various plant species against fungal pathogens. Hence, when seasonal clouds restrict sunlight, LEDs can provide a controllable, alternative source of selected single or mixed wavelength photon source in greenhouse conditions.

## 1. Introduction

Light is crucial for photosynthesis and plant growth. The effects of light on plant growth and development are complex; the entire spectrum of light is not beneficial for plants. Living organisms generally harvest the visible electromagnetic spectrum, which we will hereafter refer as “light”. Apart from photosynthesis, light also controls flowering time and morphogenesis. Two major photoreceptors—phytochromes (absorbs red/far-red-light) and cryptochromes (absorbs blue/ultraviolet A (UV-A) light)—are responsible for plant morphological and developmental changes [1,2].

**Figure 1:**
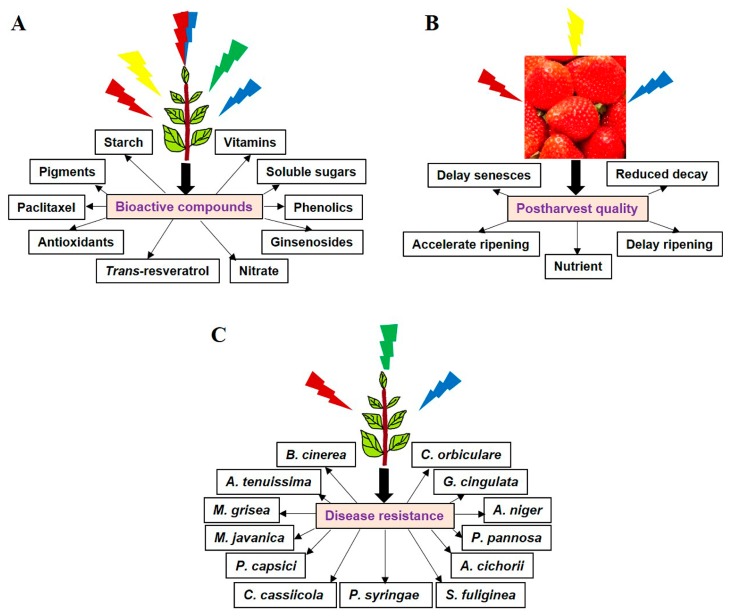
Effect of LEDs on (**A**) bioactive compounds production, (**B**) postharvest quality, and (**C**) disease resistance against different pathogens. For specific effects of LEDs on different plant traits, refer to the tables.

Various studies have demonstrated that a controlled amount of light improves the postharvest quality and shelf-life of crops, by inducing nutrients and bioactive compounds production (Figure 1) [3,4,5,6,7]. Bioactive compounds in plants are known as primary or secondary metabolites, and give aroma, color, and taste to the plants [8]. In addition, secondary metabolites provide resistance to plants against invading pathogens [8]. Numerous studies have been carried out to increase the production of bioactive compounds in plants by giving different forms of external stress [9,10,11,12]. In addition to crop sterilization, UV irradiation can also be used to induce the production of secondary metabolites [13]. Apart from UV, visible light also improves food safety and preservation, by acting as a bactericide [13,14]. High-pressure sodium (HPS), xenon, fluorescent, and incandescent lamps are the usual sources of light that are used in crop production and preservation. The use of conventional lighting systems with a broad spectrum of wavelengths may generate excessive heat and undesirable effects on plant growth and development due to inadequate protective mechanisms against UV or infrared (IR) radiations [15,16].

Nowadays, light-emitting diodes (LEDs) are emerging as a promising tool for greenhouse crop production and food preservation [15]. LEDs emit radiation within narrow bandwidths that have a relatively high photon flux or irradiance, and minimal thermal effects; additionally, LEDs can be conveniently integrated into electronic systems [17]. Altogether, such beneficial characteristics of LEDs make them very useful for agronomic purposes. Other benefits of LEDs include ambient touch temperatures and non-breakable glass envelopes; hence, they are considered to be state-of-the-art and easily-handled light sources for plant growth capable of enhancing the nutritional contents of crops [15,16,18]. LEDs are also used in postharvest preservation, due to their low heat irradiance and higher efficacy. Moreover, the role of LEDs in disease resistance makes them very useful for improving agricultural practices.

Due to the various favorable properties (i.e., robustness, compactness, and long half-life), LED lighting systems are becoming a cost-effective technology, and are ripe for adoption in the fields of agriculture and horticulture. LEDs can be implemented and tailored to the needs of the food industry as an efficient and increasingly inexpensive means of producing and dispensing satisfactory and safe foods. In this review, we focus on the potential of LEDs in the production of bioactive compounds, which boosts the quality of crops and improves crop protection. We discuss the most significant recent findings from these fields, their drawbacks, and countermeasures.

## 2. LEDs Induce Bioactive Compound Synthesis in Crops

The quality of light has a pronounced effect on the accumulation of various metabolites in plants (Figure 1A) [19]. Increased accumulation of plant metabolites, both primary and secondary (e.g., soluble sugars, starch, vitamin-C, soluble protein, and polyphenol), was observed in the presence of single-spectral red or blue LEDs when compared with white light (Table 1) [20,21,22,23,24,25]. Along with single-spectral red light, the combination of blue and red (red:blue) LEDs also increases the accumulation of primary metabolites, as well as anthocyanin, total polyphenols and flavonoids (Table 1) [26,27,28,29]. However, red LEDs have a more pronounced effect on anthocyanin accumulation than blue LEDs. This can be attributed to the increased expression of anthocyanin biosynthesis gene (i.e., *MdMYB10* and *MdUFGT*) under the influence of red LEDs [30]. Ambient light supplemented with red, blue, green, red:far-red or red:blue LEDs also elevates the accumulation of organic acids, phenolic compounds, vitamin-C, α-tocopherol, soluble sugar and nitrate in different crops (Table 1) [31,32,33,34,35,36].

LEDs’ role in the induction of secondary metabolite production in plants seems to be linked with phenylalanine ammonia-lyase enzyme (PAL), which is involved in the first step of the phenyl propanoid pathway. Hence, up-regulation of PAL, in the presence of red:blue LEDs might be responsible for increased production of plant secondary metabolites [26]. Ginsenosides are the major plant secondary metabolites produced by the isoprenoid pathway in ginseng plants (*Panax ginseng* Meyer), and which have high medicinal value. Increase in the concentration of total ginsenosides, (from 2% to 74%) in ginseng roots was noted in response to blue LEDs (450 nm and 470 nm), when compared with ginseng roots grown under dark conditions (Table 1) [51]. Therefore, it is plausible that LEDs can act as elicitors, triggering expression of key enzymes (like squalene synthase) in the isoprenoid pathway, or may also induce production of reactive oxygen species, which consequently can trigger enhanced activity of defense-related genes, thereby increasing synthesis of ginsenosides. Additionally, in red ginseng, LED exposure can spark the production of high levels of pharmacological components [51].

In different plants—including callus mass of the Himalayan yew (*Taxus wallichiana* Zucc.) tree and grapes—single-spectral blue and red LEDs play an important role in the accumulation of anticancer agents (like paclitaxel and baccatin) and also *trans*-resveratrol (which acts against cardio vascular diseases), when compared with white fluorescent light (Table 1) [52,53]. In addition to their role in increasing in paclitaxel levels, blue LEDs also play an important role in callus growth from needles and petioles explants [52]. Moreover, it has been seen that blue and red LEDs trigger changes in the synthesis of stilbene compounds in grape plants [37]. Increase in the production of stilbene compounds is a consequence of the higher expression levels of *stilbene synthase* under the influence of blue and red LEDs [37,53].

Previously, it has been hypothesized that enhanced accumulation of primary metabolites in crops could arise due to the inhibition of the translocation of photosynthetic products, caused by LEDs. Increased accumulation of secondary metabolites in response to light, including UV light, can be a stress response and/or a sun-screening effect, to protect plants from ionizing radiations [51]. Light also affects signal transduction pathways, which include enzymes, metabolites, and secondary messengers. The aforementioned evidence strongly suggests that light could be used for the production of medicinally important secondary metabolites in plants. However, the effect of different single- or mixed-spectral light ratios may vary according to the plant species or cultivars. To enhance the nutritional traits of crops, use of blue LEDs and/or combined red:blue LEDs might be the best choice, under controlled cultivation practices [6]. Nonetheless, more mechanistic investigation is required, in order to better understand how we can harness the use of LEDs for the betterment of plant developmental traits, as inconsistent responses of different metabolic pathways to varied light wavelengths pose a challenge.

## 3. LEDs Enhance Antioxidant Properties

Light quality affects the photo-oxidative properties of plants by modulating the antioxidant defense system, resulting in the rise of antioxidative enzyme activity. Enhanced antioxidant properties of many vegetables—like, pea, Chinese cabbage, kale, tomato, etc.—have been observed as a response to the use of single-spectral or combined red (625–630 mm):blue lights (465–470 mm), when compared with white light sources (Table 1) [23,24,29,38,58]. Moreover, green (510 nm), yellow (595 nm) or even mixed red:white LEDs also increase both antioxidant properties and anthocyanin accumulation (Table 1) [30,34,55]. Such improvements in the antioxidant characteristics may arise due to the induction of β-carotene, glucosinolates, free radicals (e.g., DPPH; 1,1-diphenyl-2-picrylhydrazyl), scavenging activity, ROS-scavenging enzymes (e.g., superoxide dismutase), phenolic compounds, and vitamin C [23,24,29,38,55,58]. Consuming antioxidant-rich fruits and vegetables can have health benefits. Therefore, it would be interesting to ascertain the health benefits endowed by the consumption of LED-treated crops.

## 4. LEDs Improve Nutritional Traits of the Postharvest Produce

LEDs have been used in growth chambers and greenhouses to improve plant biomass and nutrient content. Due to their energy-efficient nature, small size, long life, and relatively cool surfaces, LEDs are also used in the postharvest processing of crop produce (Figure 1B). Postharvest processing aims to maintain the desired aesthetic characteristics of the crop produce, along with farm texture, enriched nutrition, and flavor quality.

Narrow-bandwidth LEDs with different wavelengths can affect the accumulation of volatile compounds (e.g., benzenoid and phenylpropanoid) related to aroma or taste in flower and fruit products of different crops, like, petunia, tomato, strawberry, and blueberry, when compared with white light or dark growth conditions [57]. Additionally, increased levels of 2-phenylethanol (a major volatile compound) in petunia flowers has been attributed to the use of red and far-red light. This suggests that the LEDs can improve the aromatic properties of plant products in a way that can satiate our olfactory needs (human consumption) [57].

Besides enhancing the olfactory appeal, different spectral LEDs including, red, blue, green or even white light can also improve the nutritional quality of harvested vegetables, e.g., cabbage, by increasing the accumulation of vitamin C, anthocyanin and total phenolics (Table 1) [43,54]. Single-spectral blue LEDs regulate anthocyanin synthesis by up-regulating the expression of anthocyanin biosynthesis genes in Chinese bayberry fruits [46]. In addition, blue LEDs can also facilitate moisture loss by stimulating stomatal conductance and transpiration during postharvest storage of the crop produce. In contrast, red LEDs aid in moisture retention in tissues of fruits and vegetables. This may also prevent quick water loss, thereby improving their aesthetic quality and acceptability to consumers [43,58,59]. Furthermore, red or blue LEDs delays senescence of fruits by reducing the production of ethylene and ascorbates (Figure 1) [39]. As fruits are often transported through long distance, it is important to extend their shelf-life. Interestingly, blue light delays the change of color in tomatoes from green to red [45]. Moreover, tomatoes treated with blue LEDs become firm, accumulate higher levels of free amino acids, including γ-aminobutyric acid-GABA, when compared with tomatoes kept under dark condition [45]. Blue or yellow LEDs are also known to enhance ripening of fruits along with induction of the synthesis of β-carotene, lutein, α-tocopherol and γ-tocopherol when compared with fruits under dark conditions [42,47,56]. Quick ripening occurs due to the increased rates of respiration and ethylene production caused by the LEDs [42,47]. Blue light stimulates the expression of ethylene biosynthesis genes (i.e., *PpACO1* and *PpACS3*), which demonstrates the molecular mechanism of LED-mediated fruit ripening [47].

Fruit ripening is a complex developmental process governed by multiple factors, e.g., cell wall degradation and softening, cuticle thinning, and hormonal interplay. Moreover, ripening processes and the underlying molecular mechanisms are different between climacteric and non-climacteric fruits. The same LEDs may have differing impacts on the molecular processes in climacteric and non-climacteric fruits. Therefore, a detailed molecular investigation is warranted in future for complete comprehension of the effects of LEDs on the diverse postharvest crop produce.

## 5. LEDs Offer Protection against Food Spoilage and Crop Loss

Post-harvest spoilage of fruits or the protection of standing crops from the pathogen attack remains a challenge for agriculture scientists; nowadays, LEDs have been gaining attention as a handy tool for sustainable agricultural practices. For instance, single-spectral blue LEDs reduce the postharvest decay caused by *Penicillium* species in citrus fruits, when compared with dark conditions (Figure 1C; Table 2) [48,60]. Additionally, reduction in the infection of fruits has been observed due to the light-mediated stimulation of lipid signaling and subsequent accumulation of phospholipase A2, ethylene, and octanal [49,50]. Moreover, blue light can directly suppress the sporulation and germination of fungi (Table 2) [61,62,63]. Therefore, blue light-mediated post-harvest crop protection might be caused by a dual effect, resulting from the inhibition of fungal growth and stimulation of host defense responses.

Specific wavelengths of light, especially red, blue and green LEDs, can induce disease resistance in standing crops against a wide range of phytopathogens (Figure 1C; Table 2) [23,64,65,66,67,68,69,70,72,73,74,75,76]. Red light inhibits lesion development, induces expression of defense-related genes and also promotes synthesis of stilbenic compounds, when compared with such effects under white fluorescent light [37]. Stilbenes, also known as phytoalexins, play an important role in plant defense responses [77]. Moreover, increased synthesis of stilbenes, concomitant with the elevated expression of 16 defense-related genes, was observed after different wavelength exposure of plant products by LEDs [37,70]. Furthermore, LEDs can also induce the expression of defense-related genes and subsequent ginsenosides biosynthesis in Ginseng plants [78].

Salicylic acid (SA) plays a vital role in plant disease resistance. The mutants of red:far-red light photoreceptors are known to be compromised in SA signaling stimulation and resistance to *P. syringae* [79]. Red LEDs induce SA content and expression of SA-regulated *PR-1* and *WRKY* genes in pathogen-inoculated cucumber plants [69]. Taken together, it can be assumed that the red light-induced resistance may be closely associated with SA-mediated defense responses [69]. Furthermore, the low red:far-red light ratio inhibits SA and jasmonic acid (JA)-mediated disease resistance in *Arabidopsis* by reducing the expression of SA- and JA-responsive genes. This result also shows the possible effect of LEDs on SA- and/or JA-mediated disease resistance [80,81].

We know that plant defense response is quite complex—especially the crosstalk between SA and JA, and their roles against biotrophic and necrotrophic pathogens. Generally, the defense response against biotrophic and necrotrophic pathogens is mediated by SA and JA, respectively. Different spectra of LEDs can activate different molecular events which can trigger accumulation of defense hormones (i.e., SA and JA). Therefore, a comparative investigation is required to unravel the molecular response in LED-treated plants during biotrophic and necrotrophic pathogen infection.

## 6. Role of LEDs in Increasing Crop Yield

LEDs generate less heat, which enables their use as inter-lighting system under greenhouse conditions [82]. Moreover, LEDs consume less power; hence, a significant amount of energy can be saved with their use. The use of single-spectral blue or red LEDs has resulted in significant improvements in the quality and yield of vegetables and fruits (e.g., cucumber, pepper, and strawberry fruits) when compared with white fluorescent or solar light (Table 1) [31,82,83]. Moreover, LED inter-lighting systems (57 W m^−2^) accelerate the fruit maturation process [84]. Besides single-spectral light, use of mixed red:blue light can also increase the crop yield (Table 1) [31,34,83,85]. In any case, under controlled environmental conditions, red LEDs can act as a principal light source for promising growth of vegetables and to enhance the dry mass and yield (Figure 1). As blue and red light control the rates of photosynthesis through the opening and/or closing of stomata, their effect on plant biomass or yield is not surprising [85,86].

## 7. Conclusions and Future Prospects

The ultimate goal of crop production is to obtain better nutritional quality along with high yield. Because of environmental constraints and a reduction in the availability of cultivated lands, there is an urgent need to develop indoor cultivation systems in order to obtain yield parameters that are similar to or higher than outdoor cultivation systems. Conventionally, fluorescent and incandescent lamps or high-pressure sodium lamps with variable spectral emissions have been used for these purposes. However, such light sources have drawbacks, including short half-life, heat production, and high power consumption [87]. LEDs have multiple advantages over traditional light sources: ability to emit a narrow band of light, high purity and efficacy, tiny size, longer half-life, and lower power consumption [42,87]. Because of their portability, LEDs can be used in a variety of horticultural settings, such as growth chambers, greenhouse inter-lighting systems, and vertical farming [21,88]. The combination of different wavelengths of LEDs in varying proportions can improve the nutritional quality of crops or fruits either in field conditions or during postharvest processing. In addition, combination of LEDs can delay senescence of plants and vegetables, and alters ripening process in certain fruits, even under postharvest conditions. LEDs produce minimal heat, which improves food safety by inactivating foodborne pathogens in the postharvest produce. Hence, LEDs (especially blue LEDs) can be used as effective bactericides in cold storage, as bacterial growth can be inactivated more effectively at low temperatures [13,89]. LEDs may offer an alternative to chemical sanitizers to satisfy the growing global demand for food microbiological safety.

In addition to the use of LEDs in food storage, LED-induced plant disease resistance could suggest new approaches towards minimizing the use of chemicals for crop protection. Besides the use of genetically modified crops, chemical priming is an alternative approach to make plants resilient against environmental stresses. As an alternative to chemical priming, LEDs—with its eco-friendly nature—can be used as a handy tool for inducing priming. However, more research is required to determine the spectral qualities required for optimal crop protection. The technical and operational benefits of LEDs could be maximized by using the combination of desired wavelengths.

One of the major limitations of this technology, for its effective use under in vitro conditions, lies in its low penetrance. Additionally, optimal spectral conditions are not precisely known for number of crops. More greenhouse studies with different leafy vegetables could suggest new ways to use this technology in large-scale vegetable or fruit production. It would be interesting to investigate whether LEDs can be used to control the transition from vegetative to reproductive stages, depending on the plants. Narrow-band blue, red, green, or yellow light can adversely affect the vision of workers and researchers. Therefore, minimizing such issues with white light supplementations might be a future approach. For the economical deployment of LEDs, forecasting studies should assess crop-specific costs and benefits. However, several factors, including enhanced luminous efficacy of LEDs, field use efficiency, manufacturing cost, and energy consumption, will determine the future of its use.

## Figures and Tables

**Table 1:** Effect of LEDs on the synthesis of bioactive compounds and the quality of the crop produce.

LED Light	Light Intensity	Crops	Synthesis of Bioactive Compounds and Crop Traits	References
Red	50 μmol m^−2^ s^−1^	*Gossypium hirsutum*	Sucrose, starch, soluble sugar	[21]
	50 μmol m^−2^ s^−1^	*Vitis* root-stock	Sugar, starch	[27]
	80 μmol m^−2^ s^−1^	*Brassica campestris* L.	Starch	[25]
	500 μmol m^−2^ s^−1^	*Glycine*, *Sorghum*	Starch	[20]
	30 μmol m^−2^ s^−1^	*Betula* *pendula* Roth	Starch	[22]
	50–80 μmol m^−2^ s^−1^	*Vitis vinifera*	Stilbene	[27,37]
	50 μmol m^−2^ s^−1^	*Malus domestica* Borkh	Anthocyanin	[30]
	500 μmol m^−2^ s^−1^	*Triticum aestivum* L.	Lignin	[34]
	128 μmol m^−2^ s^−1^	*Pisum sativum*	β-Carotene	[38]
	50 μmol m^−2^ s^−1^	*B. oleracea* var. *italic*	Delayed senescence	[39]
Blue	100–200 μmol m^−2^ s^−1^	*Lactuca sativa*	Phenolic content, Vit-C, tocopherol, carotenoid	[24,32,36,40]
	50 μmol m^−2^ s^−1^	*Vitis* root-stock	Sugar, starch	[27,41]
	80 μmol m^−2^ s^−1^	*Brassica campestris* L.	Vit. C	[25]
	>20–40 μmol m^−2^ s^−1^	*Fragaria×ananassa*	Organic acids, anthocyanin, ripening	[31,42]
	50–80 μmol m^−2^ s^−1^	*B. rapa*, *B. oleracea* var. *capitata*	Vit. C, polyphenolic content	[25,43,44]
	85–150 μmol m^−2^ s^−1^	*Solanum lycopersicum*	Proline, Reactive Oxygen Species, scavenger activities, polyphenolic compounds, γ-aminobutyric acid, shelf-life	[23,45]
Blue	40 μmol m^−2^ s^−1^	*Myrica* *rubra* Sieb. and Zucc.	Anthocyanin	[46]
	40 μmol m^−2^ s^−1^	*Prunus persica*	Ripening	[47]
	40 μmol m^−2^ s^−1^	*Citrus reticulate*	Reduced postharvest decay	[48]
	40–630 μmol m^−2^ s^−1^	*Citrus* hybrid	Reduced pathogen infection	[49,50]
	*-*	*Panax ginseng*	Ginsenosides	[51]
	60 μmol m^−2^ s^−1^	*Taxus wallichina* Zucc	Paclitaxel	[52]
	80 μmol m^−2^ s^−1^	*Vitis vinifera*	*Trans*-resveratrol	[53]
Green	~200 μmol m^−2^ s^−1^	*Lactuca sativa*, *Lens culinaris*, *Triticum aestivum* L., *B. oleracea* var. *capitata*, *Fragaria×ananassa*	Phenolic content, Vit-C, α-tocopherol, anthocyanin	[32,36,40,43,54,55]
Yellow	~100 μmol m^−2^ s^−1^	*Raphanus sativus*, *Malus* sp., *S. lycopersicum*, *C. annuum*	Vit-C, α-tocopherol, γ-tocopherol, lutein	[55,56]
Red+Blue	70 μmol m^−2^ s^−1^	*Doritaenopsis* hort	Carotenoids, starch, sucrose, glucose, fructose	[28]
	>20 μmol m^−2^ s^−1^	*Fragaria×ananassa*	Organic acids	[31]
	90 μmol m^−2^ s^−1^	*Lactuca sativa*	Anthocyanin	[26]
	*-*	*B. rapa*, *B. alboglabra*	Polyphenol, flavonoids, glucosinolates	[29]
Red + Blue + White	210 μmol m^−2^ s^−1^	*Lactuca sativa*	Soluble sugar, nitrate contents	[35]
Red + far - red	50–200 μmol m^−2^ s^−1^	*Lactuca sativa*, *Petunia*	Phenolic content, volatile compounds	[36,57]

**Table 2:** Induced disease resistance in crops treated with different light from LEDs.

LED Light	Light Intensity	Crops	Effect on Disease	References
Red	261–550 μW/cm^2^	*Vicia faba*	Induces resistance against *B. cinerea*, *Alternaria tenuissima*	[64]
	250–287 μW/cm^2^	Rice *sl* mutants cultivar (Sekiguchi-asahi and Sekiguchi-himenomochi)	Induced resistance against *Magnaporthe grisea*	[65]
	287 μW/cm^2^	*Arabidopsis*	Induced resistance against *M. javanica, P. syringae* pv. *tomato* DC 3000	[66]
	287 μW/cm^2^	*Piper nigrum*, *Cucurbita*, *Solanum lycopersicum*	Induced resistance against *P. capsici*	[67]
	137 μW/cm^2^; 350 μmol m^−2^ s^−1^	*Cucumis sativus*	Induced resistance against *C. cassiicola* and *S. fuliginea*	[68,69]
	80 μmol m^−2^ s^−1^	*Vitis vinifera*	Induced resistance against *B. cinerea*	[37]
		*Nicotiana benthamiana*	Induced resistance against *P. syringae* pv. *tabaci*	[70]
Blue	200 μmol m^−2^ s^−1^	*Lactuca sativa*	Induced resistance against grey mold by *B. cinerea*	[40]
	50–150 μmol m^−2^ s^−1^	*Solanum lycopersicum*	Induced resistance against gray mold disease by *B. cinerea*	[23,71]
	150 μmol m^−2^ s^−1^		Suppression of sporulation of *A. cichorii*, *P. pannosa*	[61,62]
	3.4 μW/cm^2^		Reduced spore germination of *A. niger*	[63]
		*Nicotiana benthamiana*	Induced resistance against *P. syringae* pv. *tabaci*	[70]
Green	80 μmol m^−2^ s^−1^	*Fragaria×ananassa*	*Glomerella cingulate*	[72]
		*Cucumis sativus*	*C. orbiculare*, *B. cinerea*	[73]

## References

[1] Quail P.H., Boylan M.T., Parks B.M., Short T.W., Xu Y., Wagner D. (1995). Phytochromes: Photosensory perception and signal transduction. Science.

[2] Deng X.W., Quail P.H. (1999). Signalling in light-controlled development. Semin. Cell Dev. Biol..

[3] Braidot E., Petrussa E., Peresson C., Patui S., Bertolini A., Tubaro F., Wahlby U., Coan M., Vianello A., Zancani M. (2014). Low-intensity light cycles improve the quality of lamb’s lettuce (*Valerianella olitoria* [L.] Pollich) during storage at low temperature. Postharvest Biol. Technol..

[4] Costa L., Montano Y.M., Carrion C., Rolny N., Guiamet J.J. (2013). Application of low intensity light pulses to delay postharvest senescence of *Ocimum basilicum* leaves. Postharvest Biol. Technol..

[5] Glowacz M., Mogren L.M., Reade J.P.H., Cobb A.H., Monaghan J.M. (2014). High-but not low-intensity light leads to oxidative stress and quality loss of cold-stored baby leaf spinach. J. Sci. Food Agric..

[6] Kozai T., Fujiwara K., Runkle E.S. (2016). LED Lighting for Urban Agriculture.

[7] Pogson B.J., Morris S.C., Nood’en L.D. (2004). Postharvest senescence of vegetables and its regulation. Plant Cell Death Processes.

[8] Hopkins W.G., Hüner N.P.A. (2008). Secondary metabolites. Introduction to Plant Physiology.

[9] Hasan M.M., Baek K.H. (2013). Induction of resveratrol biosynthesis in grape skin and leaves by ultrasonication treatment. Korean J. Hortic. Sci. Technol..

[10] Hasan M.M., Yun H.K., Kwak E.J., Baek K.H. (2014). Preparation of resveratrol-enriched grape juice from ultrasonication treated grape fruits. Ultrason. Sonochem..

[11] Hasan M.M., Bae H. (2017). An Overview of stress-induced resveratrol synthesis in grapes: Perspectives for resveratrol-enriched grape products. Molecules.

[12] Hasan M.M., Bashir T., Bae H. (2017). Use of ultrasonication technology for the increased production of plant secondary metabolites. Molecules.

[13] Kumar A., Ghate V., Kim M.J., Zhou W.B., Khoo G.H., Yuk H.G. (2015). Kinetics of bacterial inactivation by 405 nm and 520 nm light emitting diodes and the role of endogenous coproporphyrin on bacterial susceptibility. J. Photochem. Photobiol. B Biol..

[14] Lubart R., Lipovski A., Nitzan Y., Friedmann H. (2011). A possible mechanism for the bactericidal effect of visible light. Laser Ther..

[15] Mitchell C.A., Both A.-J., Bourget C.M., Burr J.F., Kubota C., Lopez R.G., Morrow R.C., Runkle E.S. (2012). LEDs: The future of greenhouse lighting!. Chron. Hortic..

[16] Morrow R.C. (2008). LED lighting in horticulture. Hortscience.

[17] Branas C., Azcondo F.J., Alonso J.M. (2013). Solid-state lighting a system review. IEEE Ind. Electron. Mag..

[18] Yeh N.C., Chung J.P. (2009). High-brightness LEDs-Energy efficient lighting sources and their potential in indoor plant cultivation. Renew. Sustain. Energy Rev..

[19] Bian Z.H., Yang Q.C., Liu W.K. (2015). Effects of light quality on the accumulation of phytochemicals in vegetables produced in controlled environments: A review. J. Sci. Food Agric..

[20] Britz S.J., Sager J.C. (1990). Photomorphogenesis and photoassimilation in soybean and sorghum grown under broad spectrum or blue-deficient light sources. Plant Physiol..

[21] Li H.M., Xu Z.G., Tang C.M. (2010). Effect of light-emitting diodes on growth and morphogenesis of upland cotton (*Gossypium hirsutum* L.) plantlets in vitro. Plant Cell Tissue Org. Cult..

[22] Soebo A., Krekling T., Applegren M. (1995). Light quality affects photosynthesis and leaf anatomy of birch plantlets in vitro. Plant Cell Tissue Org. Cult..

[23] Kim K., Kook H.-S., Jang Y.-J., Lee W.-H., Kamala-Kannan S., Chae J.-C., Lee K.-J. (2013). The effect of blue-light emitting diodes on antioxidant properties and resistance to *Botrytis cinerea* in tomato. J. Plant Pathol. Microbiol..

[24] Johkan M., Shoji K., Goto F., Hashida S., Yoshihara T. (2010). Blue light-emitting diode light irradiation of seedlings improves seedling quality and growth after transplanting in red leaf lettuce. Hortscience.

[25] Li H.M., Tang C.M., Xu Z.G., Liu X.Y., Han X.L. (2012). Effects of different light sources on the growth of non-heading Chinese cabbage (*Brassica campestris* L.). J. Agric. Sci..

[26] Heo J.W., Kang D.H., Bang H.S., Hong S.G., Chun C., Kang K.K. (2012). Early growth, pigmentation, protein content, and phenylalanine ammonia-lyase activity of red curled lettuces grown under different lighting conditions. Korean J. Hortic. Sci. Technol..

[27] Heo J.W., Shin K.S., Kim S.K., Paek K.Y. (2006). Light quality affects in vitro growth of grape ‘Teleki 5BB’. J. Plant Biol..

[28] Shin K.S., Murthy H.N., Heo J.W., Hahn E.J., Paek K.Y. (2008). The effect of light quality on the growth and development of in vitro cultured *Doritaenopsis* plants. Acta Physiol. Plant..

[29] Lee M.K., Arasu M.V., Park S., Byeon D.H., Chung S.O., Park S.U., Lim Y.P., Kim S.J. (2016). LED lights enhance metabolites and antioxidants in chinese cabbage and kale. Braz. Arch. Biol. Technol..

[30] Lekkham P., Srilaong V., Pongprasert N., Kondo S. (2016). Anthocyanin concentration and antioxidant activity in light-emitting diode (LED)-treated apples in a greenhouse environmental control system. Fruits.

[31] Choi H.G., Moon B.Y., Kang N.J. (2015). Effects of LED light on the production of strawberry during cultivation in a plastic greenhouse and in a growth chamber. Sci. Hortic..

[32] Samuoliene G., Brazaityte A., Sirtautas R., Virsile A., Sakalauskaite J., Sakalauskiene S., Duchovskis P. (2013). LED illumination affects bioactive compounds in romaine baby leaf lettuce. J. Sci. Food Agric..

[33] Samuoliene G., Sirtautas R., Brazaityte A., Duchovskis P. (2012). LED lighting and seasonality effects antioxidant properties of baby leaf lettuce. Food Chem..

[34] Dong C., Fu Y., Liu G., Liu H. (2014). Growth, photosynthetic characteristics, antioxidant capacity and biomass yield and quality of wheat (*Triticum aestivum* L.) Exposed to LED light sources with different spectra combinations. J. Agron. Crop Sci..

[35] Lin K.H., Huang M.Y., Huang W.D., Hsu M.H., Yang Z.W., Yang C.M. (2013). The effects of red, blue, and white light-emitting diodes on the growth, development, and edible quality of hydroponically grown lettuce (*Lactuca sativa* L. var. *capitata*). Sci. Hortic..

[36] Bantis F., Ouzounis T., Radoglou K. (2016). Artificial LED lighting enhances growth characteristics and total phenolic content of *Ocimum basilicum*, but variably affects transplant success. Sci. Hortic..

[37] Ahn S.Y., Kim S.A., Yun H.K. (2015). Inhibition of *Botrytis cinerea* and accumulation of stilbene compounds by light-emitting diodes of grapevine leaves and differential expression of defense-related genes. Eur. J. Plant Pathol..

[38] Wu M.C., Hou C.Y., Jiang C.M., Wang Y.T., Wang C.Y., Chen H.H., Chang H.M. (2007). A novel approach of LED light radiation improves the antioxidant activity of pea seedlings. Food Chem..

[39] Ma G., Zhang L.C., Setiawan C.K., Yamawaki K., Asai T., Nishikawa F., Maezawa S., Sato H., Kanemitsu N., Kato M. (2014). Effect of red and blue LED light irradiation on ascorbate content and expression of genes related to ascorbate metabolism in postharvest broccoli. Postharvest Biol. Technol..

[40] Kook H.S., Park S.H., Jang Y.J., Lee G.W., Kim J.S., Kim H.M., Oh B.T., Chae J.C., Lee K.J. (2013). Blue LED (light-emitting diodes)-mediated growth promotion and control of *Botrytis disease* in lettuce. Acta Agric. Scand. Sect. B Soil Plant Sci..

[41] Poudel P.R., Kataoka I., Mochioka R. (2008). Effect of red- and blue-light-emitting diodes on growth and morphogenesis of grapes. Plant Cell Tissue Org. C..

[42] Xu F., Cao S.F., Shi L.Y., Chen W., Su X.G., Yang Z.F. (2014). Blue light irradiation affects anthocyanin content and enzyme activities involved in postharvest strawberry fruit. J. Agric. Food Chem..

[43] Lee Y.J., Ha J.Y., Oh J.E., Cho M.S. (2014). The effect of LED irradiation on the quality of cabbage stored at a low temperature. Food Sci. Biotechnol..

[44] Mizuno T., Amaki W., Watanabe H., Goto E., Hikosaka S. (2009). Effects of monochromatic light irradiation by LED on the growth and anthocyanin contents in leaves of cabbage seedlings. VI International Symposium on Light in Horticulture 907.

[45] Dhakal R., Baek K.-H. (2014). Metabolic alternation in the accumulation of free amino acids and γ-aminobutyric acid in postharvest mature green tomatoes following irradiation with blue light. Hortic. Environ. Biotechnol..

[46] Shi L.Y., Cao S.F., Chen W., Yang Z.F. (2014). Blue light induced anthocyanin accumulation and expression of associated genes in Chinese bayberry fruit. Sci. Hortic..

[47] Gong D.D., Cao S.F., Sheng T., Shao J.R., Song C.B., Wo F.C., Chen W., Yang Z.F. (2015). Effect of blue light on ethylene biosynthesis, signalling and fruit ripening in postharvest peaches. Sci. Hortic..

[48] Liao H.L., Alferez F., Burns J.K. (2013). Assessment of blue light treatments on citrus postharvest diseases. Postharvest Biol. Technol..

[49] Alferez F., Liao H.L., Burns J.K. (2012). Blue light alters infection by *Penicillium digitatum* in tangerines. Postharvest Biol. Technol..

[50] Ballester A.R., Lafuente M.T. (2017). LED Blue Light-induced changes in phenolics and ethylene in citrus fruit: Implication in elicited resistance against *Penicillium digitatum* infection. Food Chem..

[51] Park S.U., Ahn D.J., Jeon H.J., Kwon T.R., Lim H.S., Choi B.S., Baek K.-H., Bae H. (2012). Increase in the contents of ginsenosides in raw ginseng roots in response to exposure to 450 and 470 nm light from light-emitting diodes. J. Ginseng Res..

[52] Nhut D.T., Nguyen P.L.H., Don N.T., Hien N.T.T., Huy N.P., Nam N.B., Vinh B.T., Luan T.C. (2014). Induction, growth and paclitaxel content of needle-and petiole-derived calli in himalayan yew (*Taxus Wallichiana* Zucc.) under light-emitting diodes. Acta Biol. Crac. Ser. Bot..

[53] Ahn S.Y., Kim S.A., Choi S.J., Yun H.K. (2015). Comparison of accumulation of stilbene compounds and stilbene related gene expression in two grape berries irradiated with different light sources. Hortic. Environ. Biotechnol..

[54] Kanazawa K., Hashimoto T., Yoshida S., Sungwon P., Fukuda S. (2012). Short photo irradiation induces flavonoid synthesis and increases its production in postharvest vegetables. J. Agric. Food Chem..

[55] Samuoliene G., Urbonaviciute A., Brazaityte A., Sabajeviene G., Sakalauskaite J., Duchovskis P. (2011). The impact of LED illumination on antioxidant properties of sprouted seeds. Cent. Eur. J. Biol..

[56] Kokalj D., Hribar J., Cigic B., Zlatic E., Demsar L., Sinkovic L., Sircelj H., Bizjak G., Vidrih R. (2016). Influence of yellow light-emitting diodes at 590 nm on storage of apple, tomato and bell pepper fruit. Food Technol. Biotechnol..

[57] Colquhoun T.A., Schwieterman M.L., Gilbert J.L., Jaworski E.A., Langer K.M., Jones C.R., Rushing G.V., Hunter T.M., Olmstead J., Clark D.G. (2013). Light modulation of volatile organic compounds from petunia flowers and select fruits. Postharvest Biol. Technol..

[58] Muneer S., Kim E.J., Park J.S., Lee J.H. (2014). Influence of green, red and blue light emitting diodes on multiprotein complex proteins and photosynthetic activity under different light intensities in lettuce leaves (*Lactuca sativa* L.). Int. J. Mol. Sci..

[59] Massa G.D., Kim H.H., Wheeler R.M., Mitchell C.A. (2008). Plant productivity in response to LED lighting. Hortscience.

[60] D’Souza C., Yuk H.-G., Khoo G.H., Zhou W. (2015). Application of light-emitting diodes in food production, postharvest preservation, and microbiological food safety. Compr. Rev. Food Sci. Food Saf..

[61] Suthaparan A., Torre S., Stensvand A., Herrero M.L., Pettersen R.I., Gadoury D.M., Gislerod H.R. (2010). Specific light-emitting diodes can suppress sporulation of *Podosphaera pannosa* on greenhouse roses. Plant Dis..

[62] Vakalounakis D.J., Christias C. (1981). Sporulation in *Alternaria cichorii* is controlled by a blue and near ultraviolet reversible photoreaction. Can. J. Bot..

[63] Murdoch L.E., Mckenzie K., Maclean M., Macgregor S.J., Anderson J.G. (2013). Lethal effects of high-intensity violet 405-nm light on *Saccharomyces cerevisiae, Candida albicans*, and on dormant and germinating spores of *Aspergillus niger*. Fungal Biol..

[64] Islam S.Z., Honda Y., Arase S. (1998). Light-induced resistance of broad bean against *Botrytis cinerea*. J. Phytopathol..

[65] Arase S., Fujita K., Uehara T., Honda Y., Isota J. (2000). Light-enhanced resistance to *Magnaporthe grisea* infection in the rice Sekiguchi lesion mutants. J. Phytopathol..

[66] Islam S.Z., Babadoost M., Bekal S., Lambert K. (2008). Red Light-induced systemic disease resistance against root-knot nematode *Meloidogyne javanica* and *Pseudomonas syringae* pv. tomato DC 3000. J. Phytopathol..

[67] Islam S.Z., Honda Y., Sawa Y., Babadoost M. (2002). Characterization of antifungal glycoprotein in red-light irradiated broad bean leaflets. Mycoscience.

[68] Rahman M.Z., Khanam H., Ueno M., Kihara J., Honda Y., Arase S. (2010). Suppression by red light irradiation of corynespora leaf spot of cucumber caused by *Corynespora cassiicola*. J. Phytopathol..

[69] Wang H., Jiang Y.P., Yu H.J., Xia X.J., Shi K., Zhou Y.H., Yu J.Q. (2010). Light quality affects incidence of powdery mildew, expression of defence-related genes and associated metabolism in cucumber plants. Eur. J. Plant Pathol..

[70] Ahn S.Y., Kim S.A., Baek K.H., Yun H.K. (2013). Inhibiting wildfire and inducing defense-related gene expression by led treatment on *Nicotiana benthamiana*. J. Plant Pathol..

[71] Imada K., Tanaka S., Ibaraki Y., Yoshimura K., Ito S. (2014). Antifungal effect of 405-nm light on *Botrytis cinerea*. Lett. Appl. Microbiol..

[72] Kudo R., Ishida Y., Yamamoto K. (2011). Effects of green light irradiation on induction of disease resistance in plants. Acta Hortic..

[73] Kudo R., Yamamoto K., Suekane A., Ishida Y. (2009). Development of green light pest control systems in plants. I. Studies on effects of green light irradiation on induction of disease resistance. SRI Res. Rep..

[74] Khanam N.N., Ueno M., Kihara J., Honda Y., Arase S. (2005). Suppression of red light-induced resistance in broad beans to *Botrytis cinerea* by salicylic acid. Physiol. Mol. Plant Pathol..

[75] Mutar S.S., Fattah F.A. (2013). Red light-induced systemic resistance to root-knot nematodes in tomato. J. Biol. Agric. Healthc..

[76] Rahman M.Z., Honda Y., Arase S. (2003). Red-light-induced resistance in broad bean (*Vicia faba* L.) to leaf spot disease caused by *Alternaria tenuissima*. J. Phytopathol. Phytopathol. Z..

[77] Jeandet P., Douillt-Breuil A.C., Bessis R., Debord S., Sbaghi M., Adrian M. (2002). Phytoalexins from the vitaceae: Biosynthesis, phytoalexin gene expression in transgenic plants, antifungal activity, and metabolism. J. Agric. Food Chem..

[78] Ali M.B., Yu K.W., Hahn E.J., Paek K.Y. (2006). Methyl jasmonate and salicylic acid elicitation induces ginsenosides accumulation, enzymatic and non-enzymatic antioxidant in suspension culture *Panax ginseng* roots in bioreactors. Plant Cell Rep..

[79] Genoud T., Buchala A.J., Chua N.H., Metraux J.P. (2002). Phytochrome signalling modulates the SA-perceptive pathway in *Arabidopsis*. Plant J..

[80] de Wit M., Spoel S.H., Sanchez-Perez G.F., Gommers C.M.M., Pieterse C.M.J., Voesenek L.A.C.J., Pierik R. (2013). Perception of low red: Far-red ratio compromises both salicylic acid- and jasmonic acid-dependent pathogen defences in *Arabidopsis*. Plant J..

[81] Moreno J.E., Tao Y., Chory J., Ballare C.L. (2009). Ecological modulation of plant defense via phytochrome control of jasmonate sensitivity. Proc. Natl. Acad. Sci. USA.

[82] Hao X., Zheng J.M., Little C., Khosla S. (2012). LED inter-lighting in year-round greenhouse mini-cucumber production. Acta Hortic..

[83] Li H.-M., Lu X.-M., Gao Q.-H. (2016). Effects of different light qualities on the growth, photosynthetic pigments and stomatal characteristics of okra (*Abelmoschus esculentus*) seedlings. Acta Pratac. Sin..

[84] Jokinen K., Sarakka L.E., Nakkila J. (2012). Improving sweet pepper productivity by LED interlighting. Acta Hortic..

[85] Sabzalian M.R., Heydarizadeh P., Zahedi M., Boroomand A., Agharokh M., Sahba M.R., Schoefs B. (2014). High performance of vegetables, flowers, and medicinal plants in a red-blue LED incubator for indoor plant production. Agron. Sustain. Dev..

[86] Shimazaki K., Doi M., Assmann S.M., Kinoshita T. (2007). Light regulation of stomatal movement. Annu. Rev. Plant Biol..

[87] Astolfi S., Marianello C., Grego S., Bellarosa R. (2012). Preliminary investigation of LED lighting as growth light for seedlings from different tree species in growth chambers. Not. Bot. Horti Agrobot. Cluj-Napoca.

[88] Nhut D.T., Nam N.B. Light emitting diodes (LEDs): An artificial lighting source for biological studies. Proceedings of the 3rd International Conference of the Development of BME in Vietnam.

[89] Ghate V.S., Ng K.S., Zhou W.B., Yang H., Khoo G.H., Yoon W.B., Yuk H.G. (2013). Antibacterial effect of light emitting diodes of visible wavelengths on selected foodborne pathogens at different illumination temperatures. Int. J. Food Microbiol..

